# Early evidence for beer drinking in a 9000-year-old platform mound in southern China

**DOI:** 10.1371/journal.pone.0255833

**Published:** 2021-08-12

**Authors:** Jiajing Wang, Leping Jiang, Hanlong Sun

**Affiliations:** 1 Stanford Archaeology Center, Stanford University, Stanford, California, United States of America; 2 Zhejiang Provincial Institute of Cultural Relics and Archaeology, Hangzhou, People’s Republic of China; University at Buffalo - The State University of New York, UNITED STATES

## Abstract

Alcoholic beverages played an essential role in rituals in ancient societies. Here we report the first evidence for beer drinking in the context of burial ritual in early Holocene southern China. Recent archaeological investigations at Qiaotou (9,000–8,700 cal. BP) have revealed a platform mound containing human burials and high concentrations of painted pottery, encircled by a human-made ditch. By applying microfossil (starch, phytolith, and fungi) residue analysis on the pottery vessels, we found that some of the pots held beer made of rice (*Oryza* sp.), Job’s tears (*Coix lacryma-jobi*), and USOs. We also discovered the earliest evidence for using mold saccharification-fermentation starter in beer making, predating written records by 8,000 years. The beer at Qiaotou was likely served in rituals to commemorate the burial of the dead. Ritualized drinking probably played an integrative role in maintaining social relationships, paving the way for the rise of complex farming societies four millennia later.

## Introduction

Alcohol is the most widely used psychoactive agent in the world [1]. Human societies have been deeply invested in the production, consumption, and distribution of alcoholic beverages for thousands of years [2]. In Ancient Egypt, beer production was an effective mechanism that transformed agricultural produce into a value-added medium for payment and rewards, facilitating the rise of social inequalities [3]. In the Near East, ritualized feasts involving fermented beverages helped maintain social and ideological cohesion among hunter-gatherers [4–6], possibly operating as a driving force behind the initial cereal domestication [7,8]. In the Andes, *chicha* production defined social relations, structured the rhythms of agricultural activities, and provided a basis for legitimizing ruling elites [9,10]. While much research has been done to characterize the social, political, and economic roles of alcohol in ancient societies, little is known about prehistoric China, a region with a long history of alcohol production [11,12]. Previous research in this region has focused on identifying alcohol residues from the archaeological record [12,13]. The social context of alcohol consumption, however, remains understudied.

Here, we present the first evidence for beer drinking in the context of burial ritual in early Holocene southern China. Recent archaeological investigations at Qiaotou (9,000–8,700 cal. BP), a Shangshan culture site, have revealed a platform mound containing human burials, architectural remnants, fire traces, and high concentrations of painted pottery. By applying microfossil (starch, phytolith, and fungi) residue analysis to the pottery remains, we show that some of the vessels held beer made of rice (*Oryza* sp.), Job’s tears (*Coix lacryma-jobi*), and USOs. The findings suggest that beer drinking was an essential element in prehistoric funerary rituals in southern China, contributing to the emergence of complex farming societies four millennia later.

## Archaeological background

**Fig 1:**
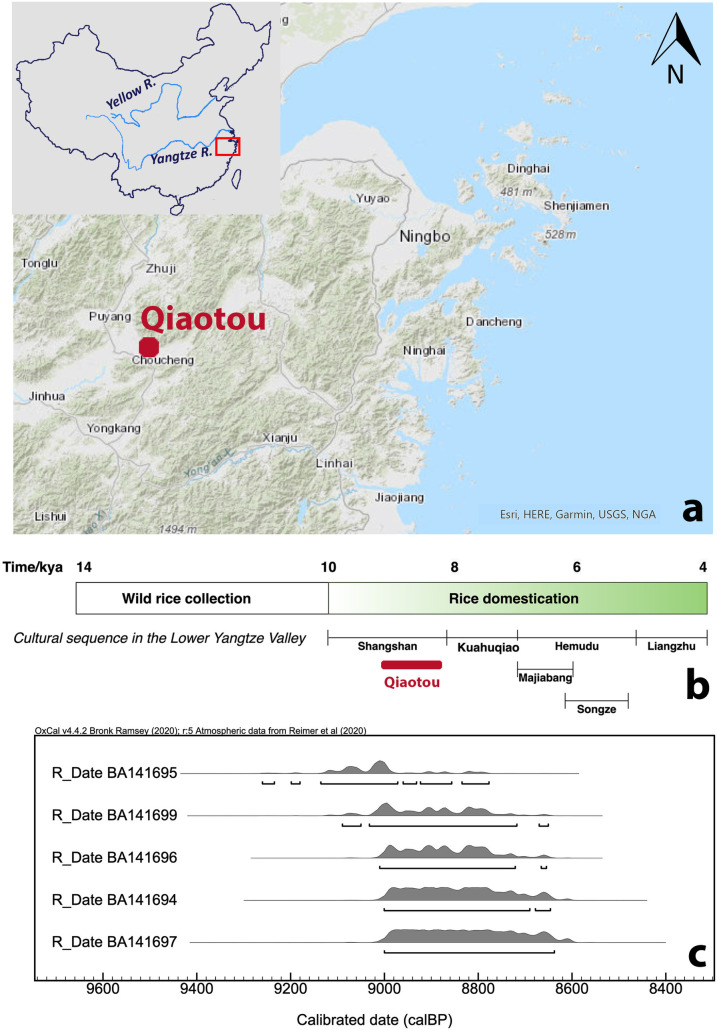
Archaeological information of the Qiaotou site. (a) Location of Qiaotou, base map modified from USGS National Map Viewer (http://viewer.nationalmap.gov/viewer/); (b) Cultural history and rice domestication in the Lower Yangtze Valley; (c) Calibrated 2σ probability distribution of AMS Radiocarbon dates on wood charcoal from Qiaotou (Peking University AMS Laboratory, calibrated by Oxcal v 4.42).

The Yangtze River Valley of southern China is the heartland of rice agriculture [14,15]. The transition from foraging to rice farming occurred gradually for about 5,000 years, from the incipient stage of rice cultivation in the early Holocene to the establishment of intensive rice farming in the Liangzhu culture (5,300–4,400 cal. BP) [16–21] (Fig 1). Shangshan was the earliest Neolithic culture in the region, capturing the onset of rice domestication and sedentism [16,17,20,22]. Recent archaeological investigations have discovered 18 Shangshan culture sites in the Jinqu Basin. In the early phase (10,000–9,000 cal. BP), settlements were relatively small villages of up to 3 ha, characterized by simple houses and pit structures. After 9,000 cal. BP, several large (size > 10 ha), ditch-enclosed settlements emerged; these sites had multi-unit dwellings, permanent storage facilities, burials, and ditches [23]. Four sites have been or are being excavated systematically, including three residential settlements, Shangshan (10,000–8,200 cal. BP), Hehuashan (9,000–8,200 cal. BP), Xiaohuangshan (ca. 9,000 cal. BP), and a non-habitation site, Qiaotou (9,000–8,700 cal. BP).

**Fig 2:**
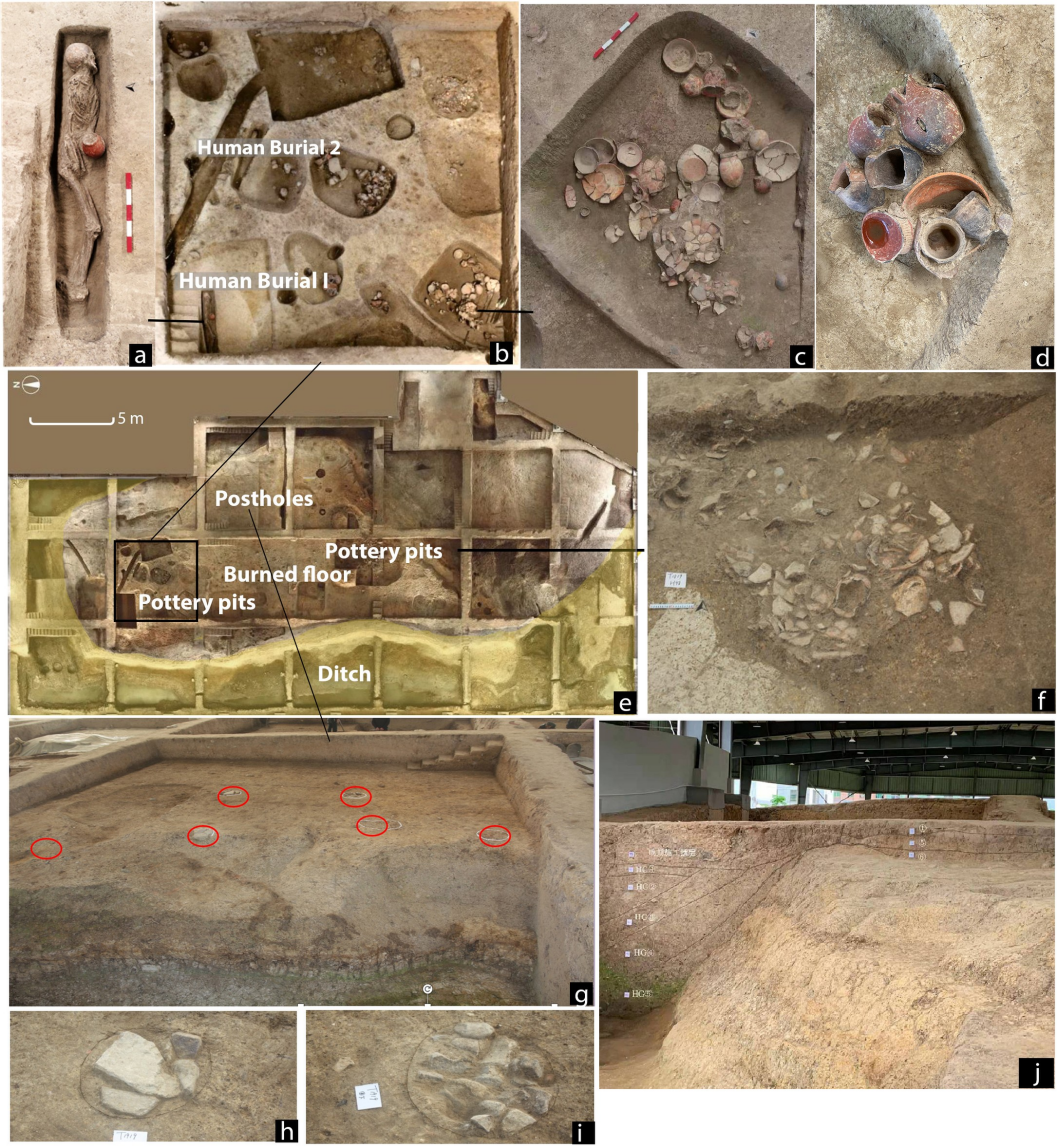
Archaeological features from Qiaotou platform mound. (a) Human burial 1(M44);b, The locations of human burials and associated pottery pits; (c) and (d) Painted pottery vessels suitable for serving foods and drinks; (e) A bird view photograph showing the location of the platform in relation to the ditch; (f) Pit H98 during excavation, which contained 50 complete pots; (g) A group of postholes from the eastern side of the platform; (h) and (i) Postholes with stone pillar bases; (j) The ditch (left) and platform (right) at Qiaotou.

Ongoing excavations at Qiaotou have revealed a platform mound (Fig 2). The mound, measuring 80 x 50 m, has an elevated surface about 3 m above ground level. It is encircled by an ancient river channel and a human-made ditch (Fig 2E), creating an enclosed compound. The ditch may have been constructed with substantial labor, measuring 10–15 m in width and 1.5–2 m in depth (Fig 2J). Two human skeletons, buried in a flexed position oriented to the east, have been found in the northern part of the platform (Fig 2A and 2B). The burials are closely associated with multiple pottery pits, some containing dense concentrations of high-quality, complete pottery vessels (Fig 2C–2F). The eastern part of the platform includes a group of postholes with stone pillar bases, which seem to be remnants of an architectural foundation (Fig 2G–2I). The artifacts recovered from the platform are dominated by elaborate pottery vessels, whereas tools associated with daily activities, such as grinding stones and harvesting implements, are relatively rare. As the current data show, the mound was likely a ritual structure rather than a residential place.

**Fig 3:**
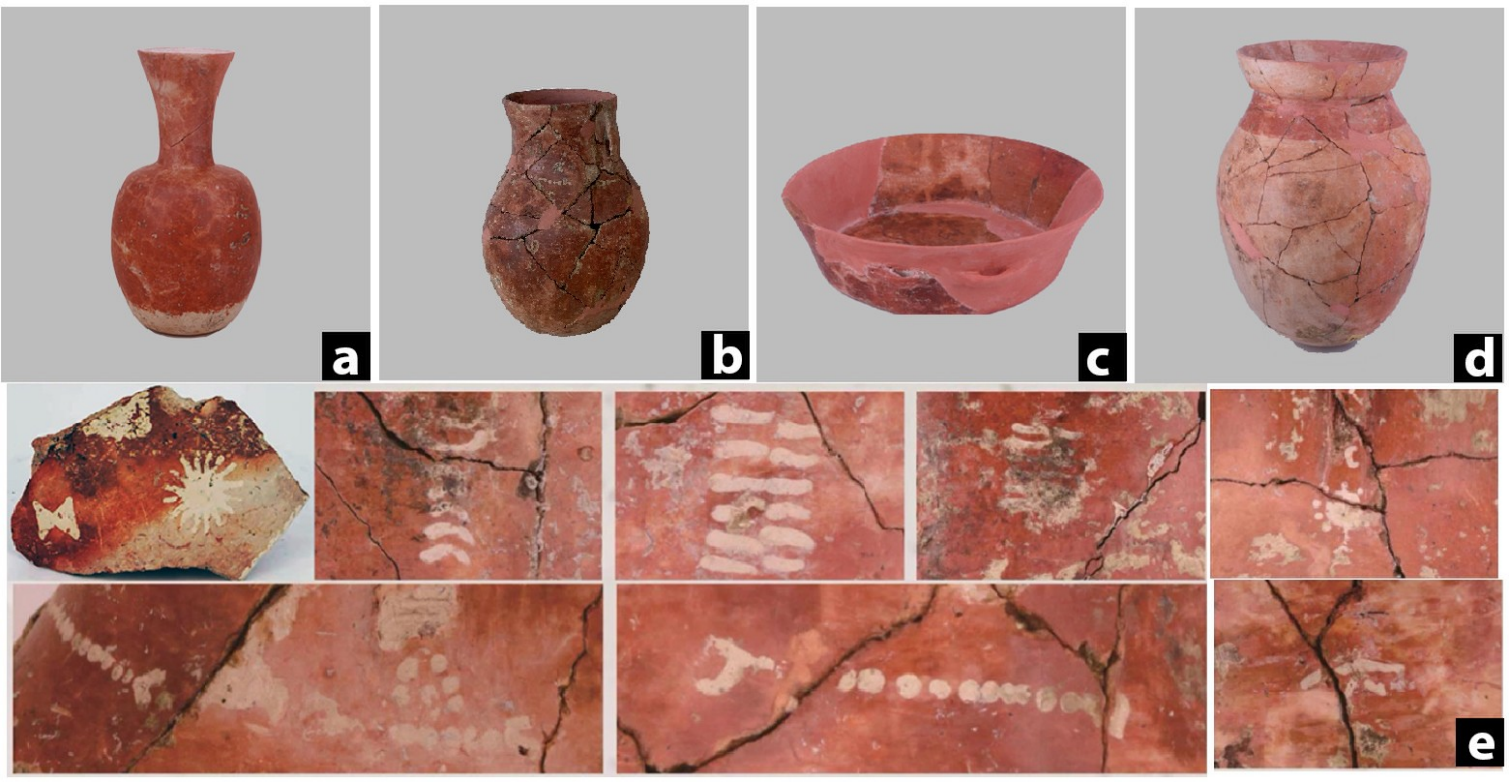
Representative pottery types recovered from Qiaotou. (a) and (b) Long-necked *hu* vessel; (c) Bowl; (d) Jar; (e) A selection of painted patterns from Qiaotou pottery.

More importantly, the pottery vessels at Qiaotou represent the earliest known painted pottery in the world [24] (Fig 3). They are slip-painted, some decorated with abstract patterns. Based on their typological characteristics, we suspect some vessels may have been alcohol containers. These vessels have narrow necks and globular bodies, akin to the bronze *hu* flask forms during the Shang and Zhou periods, a vessel type known for holding alcohol [25]. The close association between pottery pits and human burials suggests that the mound was a ceremonial place for funerary rituals involving alcohol drinking. To test this hypothesis, we analyzed 20 pots from the site.

## Materials and methods

The pottery assemblage consists of seven long-necked *hu* pots, four bowls, and nine jars. The long-necked *hu* pots are distinctive by their narrow necks, globular bodies, and slightly flaring and folded rims (Fig 3A and 3B). Bowls are flat-bottomed pots with vertical walls, some adorned with horizontal lugs and impressed decorations (Fig 3C). Jars are deep-bellied pots with flat or rounded bases (Fig 3D). All the vessels are slip-painted, with some having white dots and line decorations on their external surfaces (Fig 3E).

The vessels were unwashed and taken directly from the ongoing excavation for analysis. We extracted and analyzed three types of microfossil residues (starch, phytolith, and fungi) from interior surfaces using protocols established in the Stanford Archaeology Center (S1 Text. Methods). To rule out the potential contamination from the enclosing soil matrix, we collected seven control samples from the vessels’ exterior surfaces. Starch and phytolith identification relied on a reference collection from over 1,100 Asian economically important plant specimens and a database of fermented starch generated by our brewing experiments [26]. Fungal particles were identified according to our microbial database at Stanford and published sourcebooks [27,28]. No permits were required for the described study, which complied with all relevant regulations.

In general, beer making involves two phases: 1) saccharification, during which enzymes break down starches into fermentable sugars; 2) fermentation, during which yeasts convert sugars into alcohol and carbon dioxide. The process sometimes left diagnostic archaeological evidence, in the forms of charred cereal malts [29–31], various biomarkers [11,32,33], and modified starch residues [34]. Preservation of charred malts and biomarkers usually relies on a favorable depositional environment. Microfossils such as starch granules, on the other hand, can preserve well within the pores and cracks of ceramic artifacts. We have recently developed a methodology to identify cereal-based fermentation based on microfossil analysis [26,35]. Prehistoric brews are likely akin to a porridge that contains insoluble materials, including starches and other plant additives not fully digested during the brewing process [36,37]. These residual materials are useful for identifying alcohol-related artifacts. If a pot has been in contact with cereal-based alcohol, we would expect to find two types of microscopically observable elements. The first is starch granules showing modifications from heating and enzymatic hydrolysis during the brewing process. The second includes saccharification and fermentation agents or their sources, such as cereal malts, typical molds from fermentation starters (e.g., *Aspergillus*, *Rhizopus*, and *Mucor*), herbal plants, and yeasts. This combination of botanical and microbial elements does not exist in artifacts unrelated to alcohol or natural soils. In addition, phytoliths from cereal husks and/or herbal plants also provide information for identifying fermentation-related ingredients [12].

In our analysis of Qiaotou pottery, we applied the Congo red staining method to aid the detection of gelatinized starch [38]. After heating, starch granules absorb water and loss their regular and compact chain arrangement, allowing Congo Red to react with their amylose content. Gelatinized starch granules are stained red in bright field light, with an orange-red glow in polarized light. Unmodified starch granules do not take up the stain. Therefore, Congo Red staining is a reliable method for differentiating cooked, gelatinized starch from raw, unmodified starch.

## Results

**Fig 4:**
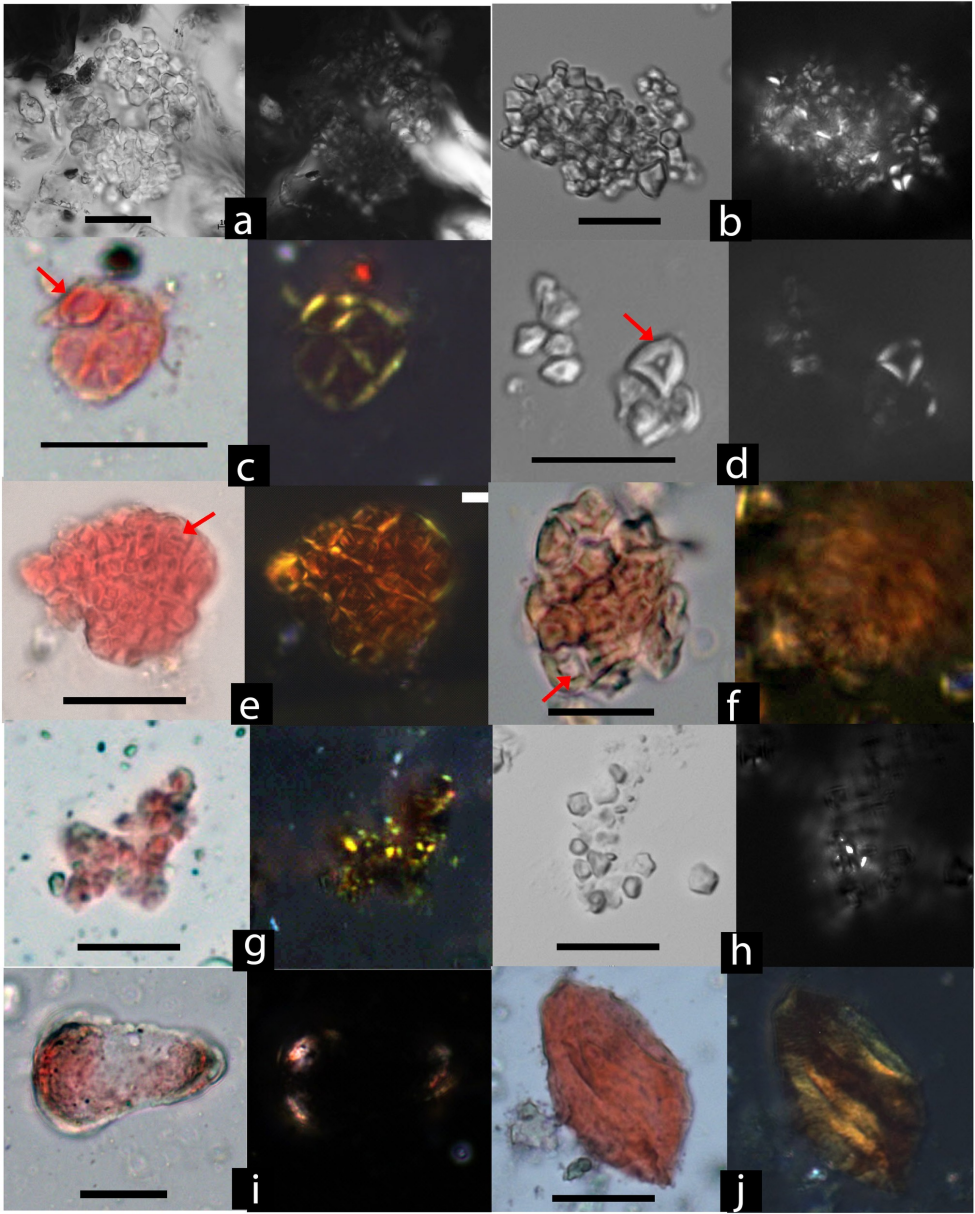
Starch granules from Qiaotou pots compared with rice brewing experiment samples. Qiaotou samples: (a) Qiaotou Type I starch (rice), compared with (b); (c) and (e) Compound rice starch granules showing central pitting (pointed by red arrows) and gelatinization (starch damage type 1), compared with (d) and (f); (g) Slightly gelatinized rice granules without pitting (starch damage type 2), compared with (h); (i) A gelatinized starch granule from Qiaotou, possibly from a USO (starch damage type 2); (j) A gelatinized starch granule from Qiaotou (starch damage type 2). Rice fermentation experiment samples: (b) A cluster of unmodified rice (*Oryza sativa*) starch granules; (c) and (f) Compound rice starch granules showing central pitting (pointed by red arrows) and gelatinization, a result of the combined effects of heating and enzymatic hydrolysis during the fermentation process; (h) Slightly gelatinized rice starch granules, showing faint extinction crosses under polarized light. Starch granules in c, e, f, h, i, and j are stained with Congo Red. They are stained red under in bright field light, with an orange-red glow in polarized light, indicating gelatinization. Each starch granule/compound is shown in bright field and polarized views (scale bars: 20 μm).

**Fig 5:**
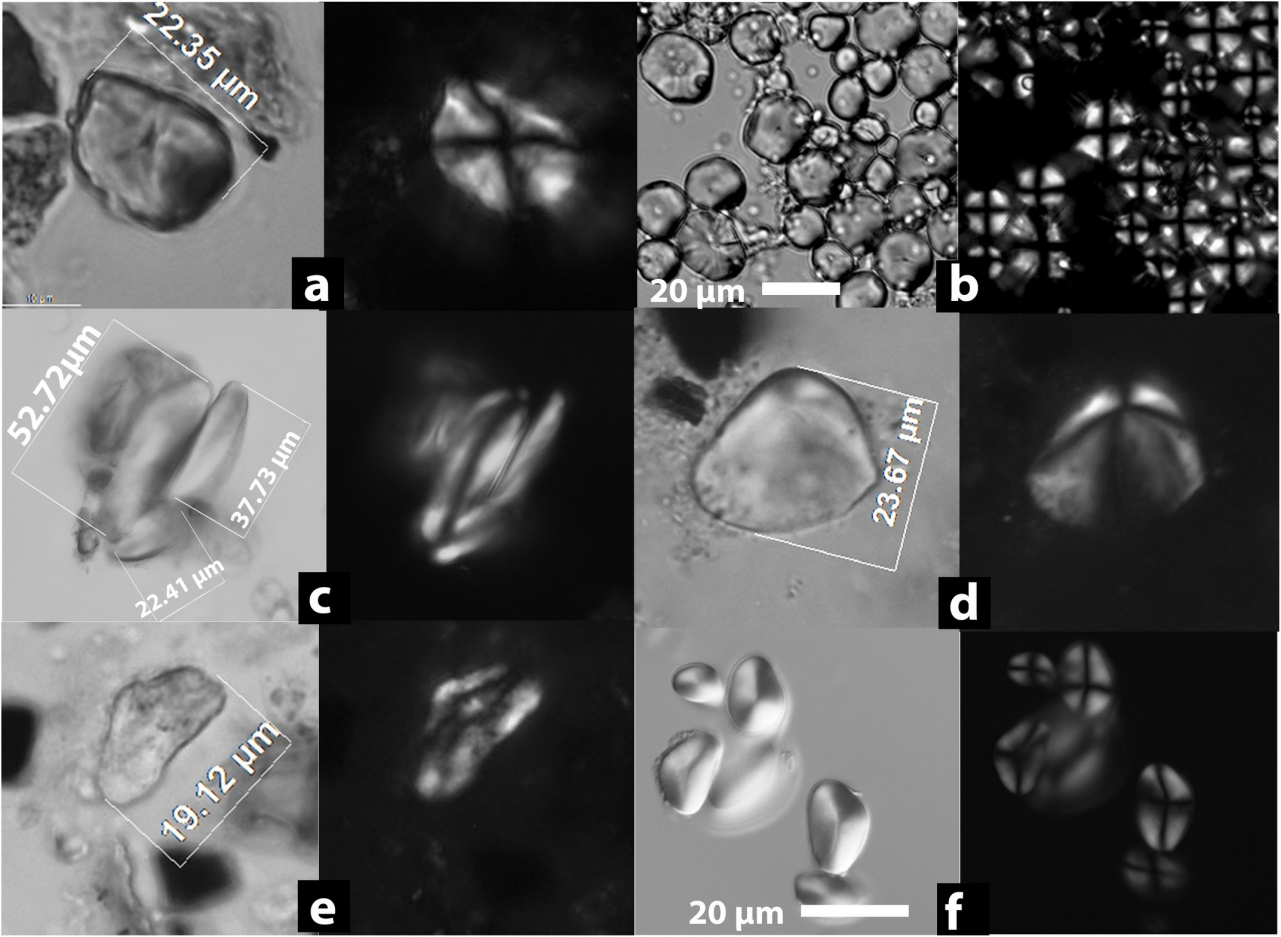
Starch remains from Qiaotou pottery compared with modern references. (a) Qiaotou Type II starch (Job’s tears); (b) Job’s tears (*Coix lacryma-jobi*, from Yunnan); (c) and (d) Qiaotou Type III starch (Unidentified USOs); (e) Qiaotou Type IV starch (acorn); (f) Acorn (*Quercus fabri*, from Zhejiang). Each starch granule/compound is shown in bright field and polarized views.

A total of 170 starch granules or compounds were recovered from Qiaotou pottery (Table 1). Of these, 95 starch granules were identifiable to various taxonomic levels when compared with our reference data. Most unidentified starch granules are gelatinized and characterized by swelling, folding, and distortion, indicating that many vessels held cooked starchy foods or drinks. Among the identified starches, rice (*Oryza* sp.) is the most ubiquitous type, followed by Job’ tears *(Coix lacryma-jobi)*, unidentified underground storage organs (USOs), and acorns (*Quercus* sp.) (Figs 4 and 5).

**Table 1:** Starch data from Qiaotou.

Artifact ID	Excavation ID	Artifact type	Rice compound	Job’s tears	UNID USO	Acorns	UNID	Total	Starch damage type 1 (fermentation)	Starch damage type 2 (cooking)	Functional interpretation
QT-POT1	T1721HG1(9)	Bowl	1			6	8	15		6	Food serving pot
QT-POT2	T1721HG1(9)H89	Bowl			1		3	4		4	Food serving pot
QT-POT3	T1721HG1(9)H89	Long-necked *hu* pot	4	3			9	16	4	3	Alcohol container
QT-POT4	T1721HG1(9)H89	Long-necked *hu* pot	3	8			11	22	3	8	Alcohol container
QT-POT5	T1721HG1(9)	Long-necked *hu* pot	1	1	1		6	9	1	1	Possible alcohol container
QT-POT6	T1721HG1(9)	Bowl	6	2	6		5	19	3	11	Alcohol container
QT-POT7	T1721HG1(9)	Jar			3			3			Undetermined
QT-POT8	T1721HG1(9)	Bowl	2		1		7	10	2	8	Alcohol container
QT-POT9	T1819(6)H98	Jar	1	2		1	4	8			Undetermined
QT-POT10	T1819(6)H98	Jar				4		4		4	Food cooking/serving
QT-POT11	T1819(6)H98	Bowl					1	1			Undetermined
QT-POT12	T1819(6)H98	Jar	1		1			2		1	Possible food serving/cooking pot
QT-POT13	T1819(6)H98	Jar		1			1	2		1	Possible food serving/cooking pot
QT-POT14	T1819(6)H98	Jar			4		5	9		5	Food serving/cooking pot
QT-POT15	T1819(6)H98	Jar				2		2			Undetermined
QT-POT16	T1819(6)H98	Jar				1	4	5		3	Food serving/cooking pot
QT-POT17	T1819(6)H98	Jar				1	1	2			Undetermined
QT-POT18	T1818(6)	Long-necked *hu* pot	2	1			3	6	1	2	Alcohol container
QT-POT19	T1817(5)H63(2)	Long-necked *hu* pot	3	3	4		3	13	2	2	Alcohol container
QT-POT20	T1716HG1(4)	Long-necked *hu* pot	7	3	4		4	18	3	1	Alcohol container
**Counts**			31	24	25	15	75	170	19	60	
**Ubiquity**			**0.55**	**0.45**	**0.45**	**0.3**	**0.8**				
Control 1	QT-POT11 exterior surface							0			
Control 2	QT-POT17 exterior surface							0			
Control 3	QT-POT18 exterior surface							0			
Control 4	QT-POT20 exterior surface							0			
Control 5	QT-POT4 exterior surface							0			
Control 6	QT-POT8 exterior surface							0			
Control 7	QT-POT19 exterior surface							0			

Type 1, rice starches (55% ubiquity; n = 31; 2.18–10.67 μm; Fig 4A), are polyhedral or round polyhedral, with centric hila and “x” shaped extinction crosses. The granules are small and appear in a compound form. Most granules are gelatinized, and their modification patterns match rice starch in our fermentation experiment (see below).

Type II, Job’s tears starches (45% ubiquity; n = 24; 10.71–22.74μm; Fig 5A), are spherical or polyhedral with two or more flat facets and centric hila that normally have T- or linear-shaped fissures. Some granules exhibit diagnostic features, including an eccentricity ratio greater than 1.47 and the presence of a Z-shaped arm on the extinction cross [39].

Type III granules are classified as unidentified underground storage organs (USOs) (45% ubiquity; n = 25; Fig 5C and 5D). They are oval, showing extremely eccentric hilum and bright extinction crosses with bent arms. However, they lack the features that would allow for identification at more specific taxonomic levels.

Type IV, acorns starches (30% ubiquity; 10.43–23.59 μm; n = 15; Fig 5E), are mostly triangular with round corners in shape. The fissure is linear and sometimes appears as a deep depression; the arms of extinction crosses are often bent, lamellae are rare, and the hila are either centric or eccentric.

Starch damage patterns provide detailed information permitting differentiation between cooked and raw plant foods. Fifteen pots revealed gelatinized starch granules, indicating that they held cooked foods and drinks. Among these, eight pots revealed microbotanical and microbial residues indicating the presence of fermented beverages. The conclusion is supported by three lines of evidence.

First, rice starch granules from these pots show morphological features consistent with our rice fermentation experiment (see [26] Fig 1 Method C and Table 2 for details). Three types of rice granules are present in Qiaotou pottery: 1) granules that show swelling with hollowed centers (starch damage type 1)(Fig 4C and 4E), resembling a diagnostic modification caused by the combined effects of enzymatic hydrolysis and heating during the brewing process (Fig 4D and 4F); 2) granules that only show gelatinization without missing parts (starch damage type 2; Fig 4G), consistent with the modifications from ordinary cooking, such as steaming (Fig 4H); and 3) unmodified granules resembling native rice starches (Fig 4A and 4B). While the first type represents the most diagnostic damage from fermentation, the latter two types are also present because starch granules within the same population may have varied responses to the same food processing technique. The residue assemblage also includes other larger gelatinized starch granules (Fig 4I and 4J), some of which are likely from Job’s tears and tubers because their unmodified starch granules are present in the same residue assemblage.

**Table 2:** Fungal elements from Qiaotou pottery and control samples.

Artifact ID	mold/mold fragment	yeast cells show budding	total
QT-POT1	0	4	4
QT-POT2	0	0	0
QT-POT3	26	2	28
QT-POT4	19	1	20
QT-POT5	18	1	19
QT-POT6	23	6	29
QT-POT7	20	12	32
QT-POT8	14	10	24
QT-POT9	0	1	1
QT-POT10	2	0	2
QT-POT11	0	1	1
QT-POT12	0	2	2
QT-POT13	0	0	0
QT-POT14	0	0	0
QT-POT15	1	1	2
QT-POT16	2	0	2
QT-POT17	1	1	2
QT-POT18	9	1	10
QT-POT19	6	2	8
QT-POT20	25	42	67
Control 1	0	0	0
Control 2	0	0	0
Control 3	0	0	0
Control 4	0	0	0
Control 5	0	0	0
Control 6	1	0	1
Control 7	1	0	1

**Fig 6:**
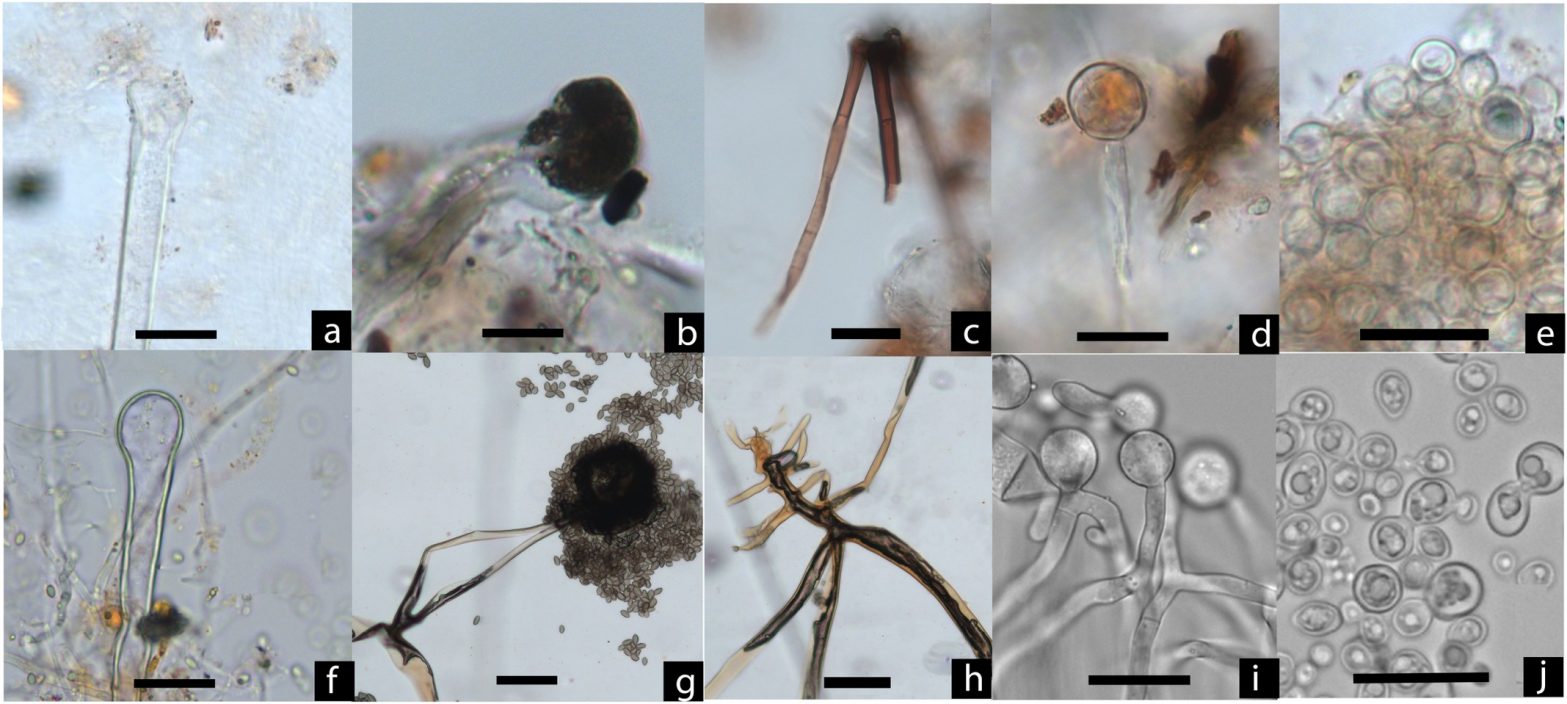
Molds and yeast cells from Qiaotou pottery compared with modern references. Qiaotou samples: (a) Vesicle/sporangia without phialides/spores attached, compared with *Aspergillus oryzae* in (f); (b) Black sporangia connecting to sporangiophores, compared with *Rhizopus* in (g); (c) *Rhizopus* sporangiophore, compared with (h); (d) Vesicle/sporangia without phialides/spores attached, compared with *Aspergillus oryzae* in (i); (e) Yeast cells in budding process, compared with (j); Modern samples: (f) *A*. *oryzae* vesicle; (g) *Rhizopus* rhizoids, sporangiophore, sporangia and sporangiospores; (h) *Rhizopus* rhizoids; (i) *A*. *oryzae* mycelium; (j) Cultured, domesticated *S*. *cerevisiae* yeast in various budding forms (scale bars: 20 μm).

Second, analyses of fungal particles (Table 2) revealed abundant fermentation-related molds and yeast cells. The molds are morphologically consistent with *Aspergillus* and *Rhizopus* (Fig 6), typical microorganisms used for making rice beer in East and Southeast Asia [40–43]. *Aspergillus* is characterized by the presence of hyaline hyphae, vesicle, and conidiophores that originate from a basal foot cell and terminate in an apical vesicle. *Rhizopus* has a body of branched mycelium composed of stolon, rhizoids, and brown sporangiophores, with a greyish-black and spherical sporangia located at the tips of the sporangiophores [27]. The yeast cells show small protuberances indicative of budding processes (Fig 6E) [44]. About 81% of the fungal elements (N = 205; 25.6/sample) were found in the pots containing fermented starch residues. The presence of specialized molds suggests that a mold starter was used for simultaneous saccharification and fermentation (see discussion and conclusion).

**Fig 7:**
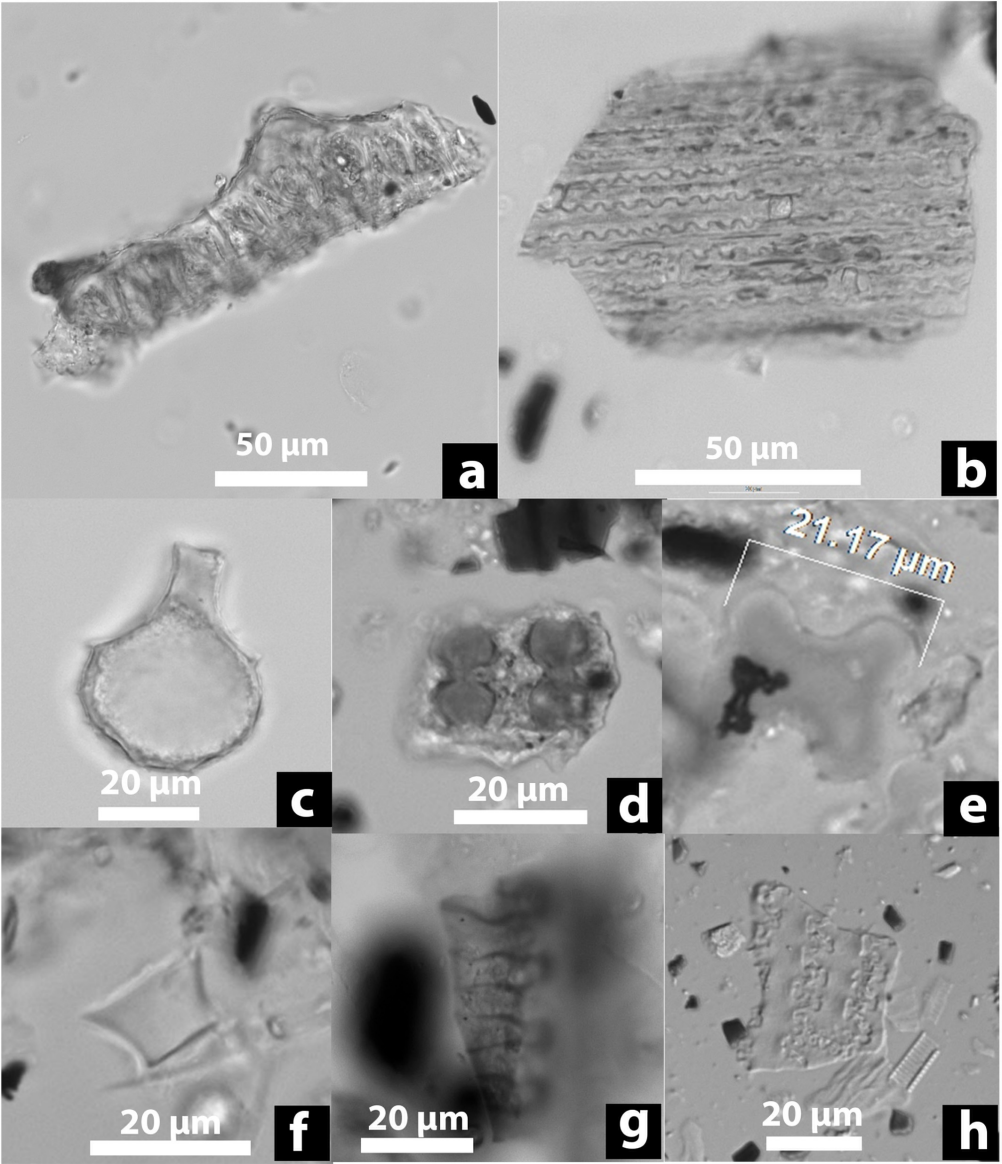
Phytolith remains from Qiaotou pottery. (a) Double peak (Oryza, rice husk); (b) elongate skeletons (Poaceae); (c) Oryza-type bulliform (Oryza, rice leaf/stem); (d) Parallel scooped bilobate (Ehrhartoideae); (f) Cross (cf. Job’s tears); (g) Rondel (Poaceae); (h) Articulated quadrilobate (Panicoideae) (i) Crenate elongate skeleton (cf. Poaceae husk).

Third, phytolith data (S1 Table, Fig 7) corroborates the starch assemblage, indicating that rice and Job’s tears were the brewing ingredients. Three types of rice phytoliths were identified, including double-peak (85% ubiquity, Fig 7A), Oryza-type bulliform (15% ubiquity, Fig 7C), and scooped parallel bilobate (30% uniquity, Fig 7D). Cross phytoliths (50% ubiquity, Fig 7E) show a considerable variation in form and size, some larger than 18μm in width, which are most comparable to the large Variant 1 cross from glume or utricle of Job’s tears [45]. Other common Poaceae family morphotypes include elongate skeletons (Fig 7B–7H), rondel (Fig 7F), and articulated quadrilobate (Fig 7G). The grass phytoliths may come from the cereal ingredients or the husks and leaves intentionally added to the brew to facilitate the fermentation process.

**Fig 8:**
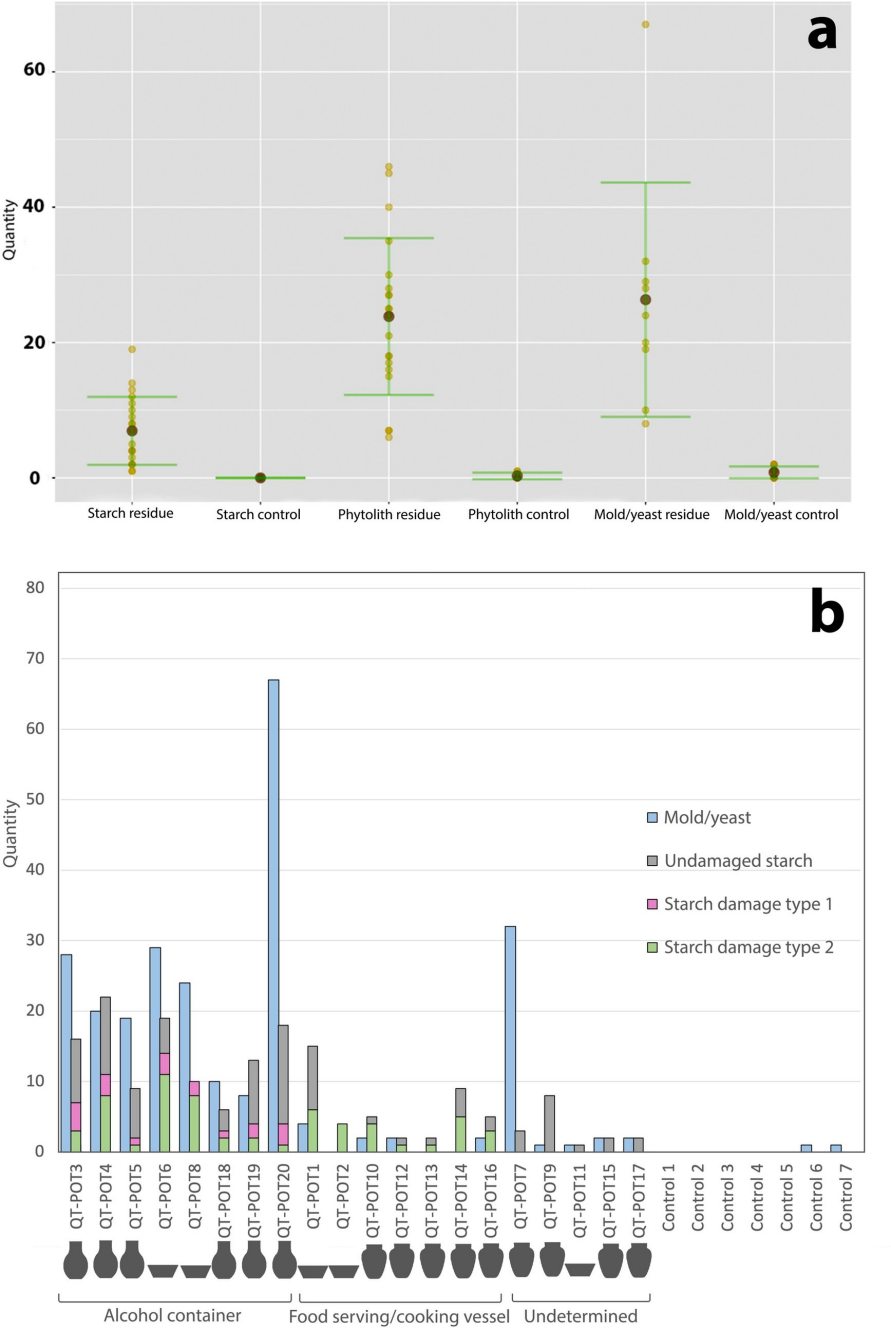
Quantitative summary of Qiaotou microfossil residue and control samples. (a) Comparison of microfossil quantities from Qiaotou residue and control samples; (b) Summary of microfossil residues from Qiaotou pottery.

We used the Mann-Whitney test to compare the starch, phytolith, and fungi counts in residue samples with control samples. The results indicate that there is a statistical significance between the two datasets (p-values < 0.05). Overall, the control samples yielded significantly lower amounts of microfossils than residue samples (Fig 8), supporting the claim that the presence of microfossil residues is the result of cultural practices associated with artifacts and rather than natural processes or contamination. In particular, the quantities of yeasts and molds are relatively small in the control samples and pots unrelated to fermentation (2.6/sample), a result in clear contrast to their abundance in fermentation-related pots (25.6/sample). This pattern indicates that most of the fungal particles in Qiaotou pottery were part of a targeted inoculation rather than later colonization during the decay of cooked foods. The only exception is POT7, a jar that contains abundant molds and yeasts but only three unmodified starch granules from a USO. Its function thus remains undetermined.

## Discussion and conclusion

### Making rice beer with a mold starter

Prehistoric alcoholic beverages include beer, wine, and mead. Wine and mead are made from fruits, honey, or other substances composed of simple sugars fermentable into alcohol with yeasts. Beer, on the other hand, is made from cereal grains or other starchy substances, such as rice, millet, barley, maize, and tubers. These raw materials contain nonfermentable starches that must be saccharified before fermentation [46]. Our analysis shows that the fermented beverage at Qiaotou was likely a beer made from rice, Job’s tears, and tubers. Starch and phytoliths from rice are the most ubiquitous in the beer-related pots (100% ubiquity), suggesting that the beverage was likely a “rice beer”.

**Fig 9:**
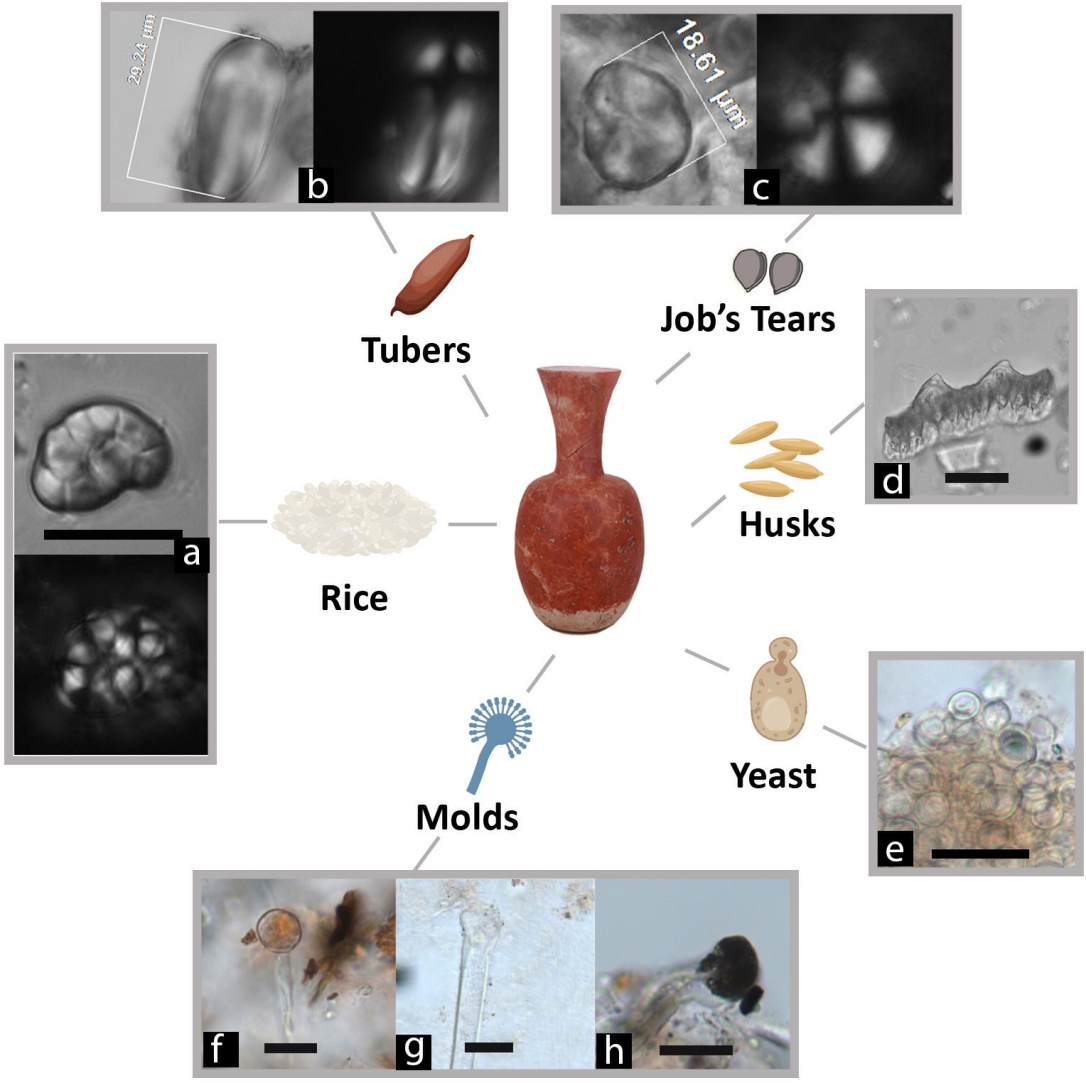
Beer-related microfossil remains. (a) Rice starch granules; (b) A starch granule from an unidentified USO; (c) A starch granule from Job’s tears, showing a characteristic Z-shaped arm; (d) A double-peak phytolith from rice husk; (e) Yeast cells in budding process; (f) and (g) vesicle/sporangia without phialides/spores attached, compared with *Aspergillus oryzae* in Fig 6; (h) Black sporangia connecting to sporangiophores, compared with *Rhizopus* in Fig 6 (scale bars: 20 μm).

According to historical records, beer making in ancient China relied on three saccharification agents: human saliva (mastication), sprouted grains (malts), and mold starters(*qu*) [47]. The mold starter method was first invented in China and later spread into other regions in Asia [48]. For example, the Khmer people in Cambodia made *Rhizopus*-rich starters by mixing rice powder with rice husks and various dried local plants [49,50]. Taiwanese indigenous peoples produced rice and millet alcoholic beverages using starters made of moldy cooked rice grains and different local herbs (e.g., *Asteraceae* and *Chenopodiaceae*) [51,52]. These herbal plants are rich in fungi and yeasts and can significantly increase the microorganism activity during brewing. If a mold starter was used for alcohol fermentation in antiquity, we would expect to find starch granules showing typical damage patterns from enzyme digestion and gelatinization due to brewing, filamentous fungi, yeasts, and grass phytoliths. All of these are indeed present in Qiaotou pottery (Fig 9).

Our findings provide the earliest evidence for using a *qu* mold starter in beer making, predating written records by at least 8,000 years [47]. The origin of the mold starter method may have been an accidental discovery: when rice grain was left in the open air in a warm and humid environment, it became moldy, producing a concentrate of fungal amylases, yeasts, and bacteria [47]. Without scientific knowledge, people at Qiaotou probably made beer by mixing cooked rice, Job’s tears, and tubers; saccharification and fermentation occurred simultaneously through the addition of *qu*, and upon further storage, a fragrant liquor was made. The Qiaotou beer appears to be different from the one discovered at the contemporary site of Jiahu in the Huai River region, which is a mixed fermented beverage made from rice, honey, and fruits [11]. It remains unclear whether honey and/or fruits were added to the brewing ingredients at Qiaotou, and this question may be addressed by chemical analysis in the future.

### The social significance of beer drinking at Qiaotou

Our analysis detected beer residues from six long-necked *hu* vessels and two bowls. The *hu* vessels are small and may have been used as individual drinking containers. The bowls are relatively large and may have been shared within a drinking party, traveling from hand to hand. At Qiaotou, large numbers of the *hu* vessels and bowls (N>20) were found in pits associated with the human burials on the platform. The discard contexts suggest that beer drinking was critical for funerary rituals.

Drinking is essentially a social act embedded with cultural and political significance [53]. The discovery of beer at Qiaotou provides comparative data for the archaeological research of social drinking in other world regions. Previous studies on this topic focused on agricultural and complex societies [3,46,54,55]. For example, research at Cerro Baúl in the Andes shows that molle beer played an essential role in organizing and legitimizing elite activities. By controlling resources for beer brewing, the Wari elites reinforced their identity and consolidated their political power [56–58]. In Chaco Canyon, drinking rituals provided a performative arena for social differentiation and claims to authority, with different forms of drinking vessels signaling social classes, ritual groups, and competitive factions [59]. In these cases, drinking and its associated paraphernalia were essential components of social competition and commensal politics.

Qiaotou adds a new dimension to the discussion by providing an early example of social drinking from a pre-agricultural, egalitarian context. Material remains from Shangshan culture settlements show little evidence of social inequality [23]. Neolithic societies in the Lower Yangtze River remained relatively egalitarian until about 6,000 BP, as indicated by mortuary and settlement data [60]. Therefore, beer drinking at Qiaotou was likely associated with other cultural and economic changes during that period. Beginning around 9,000 BP, there was a gradual trend towards more intensified practices of land modification and rice cultivation. Archaeobotanical assemblages from Huxi (9,000–8,400 cal. BP), another ditch-enclosed Shangshan culture site, show evidence of disturbed, well-lit and dry through wetlands, resembling anthropogenic habitats suitable for rice cultivation [16]. This period also witnessed the emergence of several 10 ha “mega-villages” in the region, such as Xiaohuangshan and Huxi, where multi-unit houses, deep storage pits, burials, and ditch enclosures were found together [23,61]. The construction of these settlement structures, together with intensified landscape modifications, would have required a considerable labor force. Beer drinking at Qiaotou was probably not competitive but provided opportunities for fostering social and ideological cohesion among people from different settlements.

For the Shangshan people, beer was likely a “special” or “luxury” food. Previous studies have developed a series of criteria for identifying socially valued foods in the archaeological record [62,63]. These foods are generally rare, exotic, expensive to procure and process, symbolically potent, and valued for their taste or other qualities. The data from Qiaotou fulfill some of the criteria. First, the material remains at Qiaotou suggest that the site was a special and possibly high-status place. Unlike the coarse and mostly undecorated pottery from the residential sites, the vessels at Qiaotou are of high quality, characterized by their fine materials, thin walls, and painted surface decorations. These vessels may have been used exclusively for ritual paraphernalia. Second, the pattern of plant use at Qiaotou is different from that of the earlier and contemporaneous residential sites. Analyses of grinding stones and pottery from Shangshan and Hehuashan suggest that acorns and USOs were the main staple foods, and rice was a minor component in the subsistence economy [64,65]. The ubiquity of rice remains at Qiaotou suggest that it was a luxury crop reserved for special events. Third, like many other fermented beverages, beer is an inebriant with the potential to produce a distinctive suite of physiological and psychological effects [63]. These effects would make beer highly valued and critical for social gatherings [66,67]. Finally, rice harvesting and processing may have been a labor-intensive task. Experimental studies [68,69] show that a prehistoric forager would have to spend more than eight hours on wild rice gathering to obtain adequate daily food. In contrast, collecting other resources, such as shellfish, yam, or bamboo roots, would take only about two to three hours. During the Shangshan period, rice was in the early stage of domestication; its acquisition and production probably involved high labor costs [17,22].

Since the so-called “Braidwood Symposium” in 1953, the consumption of alcoholic beverages has been recognized as a possible driving force in cereal cultivation and the transition to agriculture. The “beer hypothesis,” which was first proposed by Sauer [7] and supported by Katz and Voigt [8], has gained increasing evidence from recent archaeological data. Possible evidence of beer has been identified at the Raqefet cave (13,700–11,700 cal. BP), a Natufian graveyard in Mt. Carmel, Israel, as well as Göbekli Tepe in southeastern Turkey, a monumental sanctuary erected by hunter-gatherer groups [5,6,70]. The discovery of rice beer at Qiaotou provided supporting evidence for the “beer hypothesis.” It is important to note that, however, rice domestication had been ongoing for about a millennium before Qiaotou was constructed [17,22]. Beer drinking may have been one of many contributing factors in the protracted process of rice domestication. Future research from other Neolithic sites in southern China will elucidate this issue more clearly. Overall, the current data suggest that Qiaotou was an early Neolithic ritual center. Ritualized drinking may have provided incentives for cooperative action in early Neolithic southern China, paving the way for the development of complex rice farming societies that emerged four millennia later.

## Supporting information

S1 Table Phytolith data from Qiaotou pottery. (PDF)

S1 Text Methods. (PDF)
