# Supplementary material for: Early evidence for beer drinking in a 9000-year-old platform mound in southern China
Source: PLoS One. 2021 Aug 12;16(8):e0255833. doi: 10.1371/journal.pone.0255833 (PMC8360526; doi:10.1371/journal.pone.0255833)
Supplement: S1 Table — (PDF) [file pone.0255833.s001.pdf]

S1 Table. Phytolith data from Qiaotou pottery.

	**Silica skeletons**			**Single-cell phytolith**												
	Articulated quadrilobate	Elongate Echinate/Crenate/Dendriform	Elongate Psilate/sinuate	Double-peak	Oryza type bulliform	Scooped bilobate parallel	Scooped bilobate	Bilobate	Polylobate	Cross	Saddle	Rondel	Common bulliform	Elongate psilate/sinuate	Globular echinate	Total
QT-POT1			1	15	1					1	3		2	4		27
QT-POT2	2							8		1			1	12		24
QT-POT3				10				4	1			2	1			18
QT-POT4				4	3	3		14		3	1		7	11		46
QT-POT5		1		1				2		4	1	1		6	1	17
QT-POT6		1		7				2				1		4		15
QT-POT7				5	1			2			1			7		16
QT-POT8		3	2	1			3		11			1		5		30
QT-POT9				1			1				3	1	1	9		18
QT-POT10		1			1	1							1	2		6
QT-POT11			2	23		2				3				5		35
QT-POT12		1		2			1	3	1	12				5		25
QT-POT13			1	3									1	2		7
QT-POT14				15				2		2				2		21
QT-POT15	1	2	1	20		2		2	1	3	2		2	4		40
QT-POT16			2	7			1	7		8				3		28
QT-POT17				4				2		1						7
QT-POT18			3	6		2	6	2	3					5		27
QT-POT19			1	4		2	2	22		9				5		45
QT-POT20		1		15									3	6		25
Ubiquity	0.1	0.35	0.35	0.85	0.15	0.3	0.3	0.75	0.25	0.5	0.25	0.25	0.4	0.85	0.05	
Control 1														1		1
Control 2																
Control 3																
Control 4														1		1
Control 5																
Control 6																
Control 7																
