# Supplementary material for: Early evidence for beer drinking in a 9000-year-old platform mound in southern China
Source: PLoS One. 2021 Aug 12;16(8):e0255833. doi: 10.1371/journal.pone.0255833 (PMC8360526; doi:10.1371/journal.pone.0255833)
Supplement: S1 Text — (PDF) [file pone.0255833.s002.pdf]

Supplementary Information Text. Methods

Field sampling methods and controls for contamination

The artifacts were unwashed prior to residue extraction and taken directly from ongoing excavation for analysis. Only new and sterile plastic bags, test tubes, pipettes, toothbrushes and razor blades were used during the sampling process. The procedure used is as follows.

First, all artifacts were washed in running water for 1 min. This step helped to remove the contamination from surface sediment. After washing, all artifacts were subject to sonication for residue extraction. Each artifact was placed in a new polyvinyl bag. The interior surface of the artifact was then brushed gently with an ultrasonic toothbrush and at the same time a pipette was used to add distilled water to rinse the surface. Only a new ultrasonic toothbrush and a new pipette were used for each artifact. The water and all sediment from the bag were transferred into a new 15-ml test tube. Each tube was stored in a sealed plastic bag before laboratory analysis.

Second, in order to eliminate potential contamination from the post-depositional process, seven control samples were collected from the exterior surface of artifacts. The sampling procedure was the same as that for residue samples.

Laboratory techniques for microfossil analyses

The protocol for microfossil extraction is as follows:

i) Sample Concentration. Each 15 mL tube containing sediment and water was topped off with distilled water and placed in a centrifuge for 5 min at 1,500 rpm to concentrate the sample at the bottom of the tube. The supernatant was then decanted.

ii) Dispersion. 4 mL of 0.1% EDTA ($Na_2EDTA \cdot 2H_2O$) solution was added to each tube. Then the capped tubes were placed in an automatic shaker for 2 h to disperse the sediments. After being removed from the shaker, the tubes were filled to 15 mL with distilled water and centrifuged for 5 min at 1,500 rpm, and the supernatant was decanted.

iii) Heavy liquid separation. 4 mL of heavy liquid SPT (sodium polytungstate) at a specific gravity of 2.35 was added to the tubes. The tubes were then centrifuged for 15 min at 1,000 rpm. The top 1-2 mm layer of organics was carefully removed from each test tube by a new pipette and then transferred into a new 15 mL tube. The samples were topped off with distilled water and centrifuged for 5 min at 1,500 rpm to concentrate the starch and phytolith at the bottom of the tube, and the supernatant was decanted. The rinse was repeated two more times to remove any remaining SPT.

iv) Slide mounts and microscope scanning. An aliquot of residue sample was extracted with a pipette to a microscope slide. Congo red (0.1%, 1mg/ml) method (1) was used to dye 14 samples from Qiaotou to help determine the presence of gelatinized starch granules, which show red color under the bright field and golden glow under polarized light. Then the residue was resuspended in 30-40 μL of 50% glycerol. A coverslip was placed on top, and the edges were

sealed with nail polish. All slides were scanned under a Zeiss Axio Scope A1 fitted with polarizing filters and differential interference contrast (DIC) optics, at 200× and 400× for both starch and phytoliths. Images were taken using a Zeiss Axiocam Hrc3 digital camera and Zeiss Axiovision software Version 4.8.

SI Reference

1. Lamb J, Loy T. Seeing red : the use of Congo Red dye to identify cooked and damaged starch grains in archaeological residues. Journal of archaeological science. 2005;32(10):1433–40.
